# Central and Peripheral Cannulation for Cardiopulmonary Bypass in Fetal Sheep: A Comparative Study

**DOI:** 10.3389/fcvm.2021.769231

**Published:** 2021-12-13

**Authors:** Yun Teng, Miao Tian, Bingxin Huang, Wentao Wu, Qiuping Jiang, Xiaokang Luo, Wei Pan, Jian Zhuang, Chengbin Zhou, Jimei Chen

**Affiliations:** ^1^Department of Cardiovascular Surgery, Guangdong Cardiovascular Institute, Guangdong Provincial People's Hospital, Guangdong Academy of Medical Sciences, Guangzhou, China; ^2^Guangdong Provincial Key Laboratory of South China Structural Heart Disease, Guangzhou, China

**Keywords:** critical congenital heart disease, cardiopulmonary bypass, cannulation, comparative study, fetal cardiac surgery

## Abstract

**Objective:** *In-utero* correction is an option for treatment of critical congenital heart diseases (CHDs). Fetal cardiac surgery for CHDs is dependent on the reliable use of fetal cardiopulmonary bypass (CPB), but this technology remains experimental. In this study, we established fetal CPB models with central and peripheral cannulation to explore the differences between the two cannulation strategies.

**Methods:** Ten fetal sheep with 90–110 gestational days were randomized into central cannulation (*n* = 5) and peripheral cannulation (*n* = 5) groups. All fetal CPB models were successfully established. At each time point (0, 30, and 60 min after initiation of CPB), echocardiography was performed. Blood samples were also collected for blood gas analysis and tests of myocardial enzymes and liver and kidney function.

**Results:** In the central cannulation group, right ventricular Tei index significantly increased (*p* = 0.016) over time. Compared with the peripheral cannulation group, the left ventricular Tei index of the central cannulation group was significantly higher (1.96 ± 0.31 vs. 0.45 ± 0.19, respectively; *p* = 0.028) and the stroke volume was lower (0.46 ± 0.55 vs. 2.13 ± 0.05, respectively; *p* = 0.008) at 60 min after CPB. Levels of liver and kidney injury markers and of acid-base balance, including alanine aminotransferase (ALT), aspartate aminotransferase/ALT ratio, blood urea nitrogen (BUN), BUN/creatinine ratio, base excess and bicarbonates, were significantly higher for peripheral than for central cannulation. Other important physiologic parameters, including heart rate, blood pressure, myocardial enzymes, umbilical artery beat index and resistance index, left ventricular Tei index, and left and right ventricular stroke volume, were comparable between the two groups.

**Conclusions:** Both central and peripheral cannulations can be used to establish fetal CPB models. Central cannulation causes more adverse impacts for cardiac function, whereas peripheral cannulation is more susceptible to complications related to inadequate organ perfusion.

## Introduction

Complex congenital heart disease (CCHD) is a major cause of infant death worldwide. For many patients with critical CCHDs, *in utero* correction of the structural malformation of the heart can be used to improve treatment success. At present, the treatment of many non-cardiac fetal conditions (such as neural tube defects and twin-twin transfusion syndrome) through fetoscopy or an open-chest approach can achieve satisfactory outcomes (1–3), however, current treatment options for fetal CCHD are still limited (4). Technological advances in the past decade have enabled the development of *in-utero* cardiac intervention that can partially alleviate disease severity and reduce postnatal mortality for some critical CCHDs, including pulmonary atresia with intact ventricular septum, aortic stenosis with evolving left heart hypoplasia, and hypoplastic left heart with restrictive foramen ovale (5, 6). However, fetal cardiac intervention is only indicated for a limited number of conditions, and the majority of fetuses diagnosed with CCHD have to wait for assessment and surgery after birth.

Fetal cardiac surgery is considered the most effective method to treat fetal cardiac lesions, but this field is still in its infancy and has not been widely adopted in clinical practice due to the immature technology of fetal cardiopulmonary bypass (CPB). Bradley et al. (7) reported the first experimentation of CPB in fetal sheep, which are the preferred experimental animals (8). A conventional fetal sheep CPB model is established with median sternotomy and cannulation of the main pulmonary artery and right atrial appendage (i.e., central cannulation) (9–11). The use of the peripheral artery and vein for cannulation (i.e., peripheral cannulation) to build a model has also been reported in a few studies (12–15). Since the pros and cons of different cannulation strategies are not clearly identified, the ideal cannulation strategy to establish a fetal sheep CPB model remains to be determined.

In this study, we have established fetal CPB models with two cannulation strategies. We explored differences between central and peripheral cannulation by comparing changes in cardiac function, hemodynamics, placental function and organ perfusion after a fetal CPB procedure.

## Methods and Materials

### Study Animals

A total of ten small-tail Han sheep of 90–110 days gestation were randomly assigned to the central or peripheral cannulation groups. All animals were fed *ad libitum*. The breeding facilities had adequate ventilation, with a temperature of 20–25°C and a relative humidity of 50–70%.

### The Fetal CPB Model

All animals were fasted for 12 h before surgery. After intramuscular injection of xylazine hydrochloride (0.1 mL/kg), a tube was inserted into the deep jugular vein, and propofol (4–6 mg/kg) was continuously injected to maintain general anesthesia. The animals were intubated and ventilated, with vital signs being monitored on an electrocardiogram (ECG) display. A pregnant sheep was placed in a supine position and was examined by abdominal ultrasound to assess the orientation of the fetal sheep and determine the position of the head. The weight of the fetus was estimated by ultrasonographic assessment (16). After disinfection, a midline incision was made on the lower abdomen to expose the uterus. The positions of fetal sheep's head and sternum were confirmed by touching the uterus, and the uterine wall was incised near the fetal sheep's sternum. The amniotic cavity was first opened with a 2 cm incision to determine the position of the trunk and limbs, and the incision was gradually extended for complete exposure of the surgical field. One side of the upper limb and axillary artery were exposed for cannulation to continuously monitor blood pressure.

**Figure 1:**
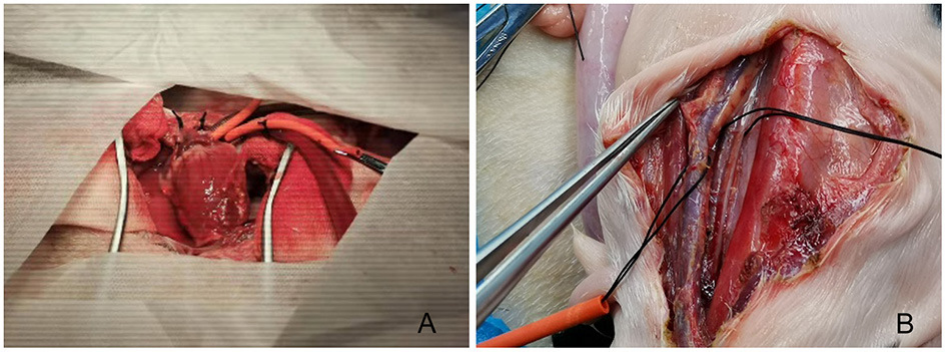
Fetal sheep cardiopulmonary bypass model established with central **(A)** or peripheral **(B)** cannulation.

For animals in the central cannulation group, one side of each of the upper limbs of the fetus was immobilized with gauze soaked in warm water, and the head was protected with thick gauze pads. A median sternotomy was performed, along with the use of bone wax and electrocoagulation to stop bleeding. Parts of the pericardium corresponding to the area between the right ventricle and main pulmonary artery were incised longitudinally, with the lower half remaining intact to restrain the left ventricle from protruding and upturning. After lifting the pericardium, the main pulmonary artery and right atrium were exposed. A purse-string suture was placed on the main pulmonary artery using a 7-0 prolene, after which the artery was cannulated with a 6 Fr straight-tip arterial cannula (Medtronic, Minneapolis, MN, USA.). A 7-0 prolene suture with a pericardium buttress was used for a purse-string suture in the right atrial appendage, and a 12 Fr straight-tip venous cannula (Medtronic, Minneapolis, MN, USA) was inserted (Figure 1A).

For animals in the peripheral cannulation group, the fetal sheep's head was turned to one side after the uterus was opened to expose the area near the jugular veins. A longitudinal skin incision along the blood vessel was made to expose the carotid artery and jugular vein. After placing a purse-string suture with 7-0 prolene, the carotid artery and the jugular vein were cannulated separately (6 Fr straight-tip arterial cannula, Medtronic, Minneapolis, MN, USA), and the venous cannula (12 Fr straight-tip venous cannula, Medtronic, Minneapolis, MN, USA) was adjusted under the guidance of ultrasound to the middle of the right atrium to complete the setup for CPB (Figure 1B).

During cannulation, animals were heparinized with heparin sodium (3–5 mg/kg, 375–625 IU/kg). After connection of arterial and venous cannulas to a standardized set of extracorporeal tubing for infants (Medos Medizintechnik AG, Stolberg, Germany), a centrifugal pump (Revolution 5 Sorin Group Italia s.r.l., Mirandola, Italy) was used to establish a normothermic CPB model without heart arrest. An extracorporeal membrane oxygenation (ECMO) oxygenator (HILITE 800LT, Medos Medizintechnik AG, Stolberg, Germany) and the placenta were both used for oxygenation. Air was used as the source of gas for the oxygenator to simulate the physiological hypoxic state of fetal sheep (PaO_2_, 30–35 mmHg). The hypoxic state was monitored by blood gas analysis (Blood Gas and Chemistry Analyzer i15, EDAN Instruments, Inc. Shenzhen, China). The priming solution was composed of 150–200 mL blood from the pregnant sheep, 10 mg (1,250 IU) of heparin and 5–10 mL of sodium bicarbonate (5%). Sodium bicarbonate was added during the subsequent CPB procedure to adjust blood acid-base balance based on blood gas measurements. The alpha-stat strategy was used during CPB management. Volume was dynamically adjusted to maintain a flow of 150–200 mL/kg/min during CPB.

### Echocardiographic Assessment and Biochemical Analysis of Blood Samples

The fetal sheep was examined by echocardiogram at the beginning (T0), 30 min (T1), and 60 min (T2) after initiation of CPB. Several echocardiographic parameters were collected, including left ventricular isovolumic contraction time (LV-IVCT), left ventricular isovolumic relaxation time (LV-IVRT), right ventricular isovolumic contraction time (RV-IVCT), right ventricular isovolumic relaxation time (RV-IVRT), aortic valve diameter (AVD), left ventricular outflow tract velocity time integral (LVOT-VTI), pulmonary valve diameter (PVD), right ventricular outflow tract velocity time integral (RVOT-VTI), umbilical artery pulsation index (Umbilical artery-PI), and umbilical artery resistance index (Umbilical artery-RI). The left and right ventricular Tei Index were calculated with the formula: [(IVCT-IVRT)/IVRT] (17). The left ventricular stroke volume (SV-LV) was derived from the formula: [LVOT-VTI × π × (AVD/2) 2]. The right ventricular stroke volume (SV-RV) was derived from the formula: [RVOT-VTI × π × (PVD/2) 2] (18).

Serum samples were collected at T0, T1, and T2 for myocardial enzyme analysis, including cardiac troponin I, myoglobin, N-terminal pro B type natriuretic peptide, creatine kinase and D-Dimer. Venous serum samples were also collected for blood biochemical tests, including alanine aminotransferase, aspartate aminotransferase, total serum protein, serum albumin, globulin, amylase, total cholesterol, serum total bilirubin, total calcium, blood urea nitrogen, creatinine, and serum phosphorus. Arterial blood samples of fetal sheep were collected at T0, T1, and T2 for blood gas analysis, including pH, partial pressure of oxygen, partial pressure of carbon dioxide, lactic acid, base excess in the extracellular fluid compartment, base excess, hematocrit, actual bicarbonate, standard bicarbonate, and oxygen saturation.

### Statistical Analysis

The measurement data were expressed as mean ± standard deviation for normal data and as median and interquartile range for non-normal data. Count data were expressed as a ratio or percentage when appropriate. Repeated measures ANOVA were used to test the difference between groups for normal data collected at different time points. Non-parametric tests were used for comparison of non-normal data. A value of *p* < 0.05 was considered statistically different. All analysis was performed using IBM SPSS Statistics 25.0 software (Armonk, New York, United States).

## Results

Fetal sheep CPB models were successfully established in a total of 10 animals. After the experiment, all pregnant sheep received hysterectomies and were extubated after recovery from anesthesia. In the central CPB group, the mean weight of pregnant sheep was 49.3 ± 1.4 kg, and the mean weight of fetal sheep was 2.1 ± 0.6 kg. In the peripheral CPB group, the mean weight of pregnant sheep was 46.5 ± 0.7 kg, and the mean weight of fetal sheep was 2.0 ± 0.4 kg. The mean gestational age of the fetal sheep in the central and peripheral groups was 103.4 ± 3.6 days and 100.4 ± 10.2 days, respectively.

The changes measured for levels of myocardial enzymes are shown in Table 1. The levels of troponin, myoglobin, NT-proBNP, creatine kinase and D-Dimer in the peripheral cannulation group were not statistically different from those in the central cannulation group (*p* > 0.05), indicating a similar extent of myocardial injury. When compared across time points within each group, cTnI levels in the central group remained stable during the entire procedure. However, the level of cTnI in the peripheral group was significantly elevated at T2 compared with that at T0 (*p* = 0.007) and T1 (*p* = 0.026).

**Table 1:** Myocardial enzyme and D-dimer levels.

	**Central cannulation**			**Peripheral cannulation**			*P*-value^*^
	**T0**	**T1**	**T2**	**T0**	**T1**	**T2**	
cTnI (ng/mL)	0.67 ± 0.41	0.49 ± 0.27	0.53 ± 0.36	0.09 ± 0.10	0.27 ± 0.218	0.87 ± 0.864	0.254
Myo (ng/mL)	<5.0	<5.0	<5.0	<5.0	<5.0	<5.0	-
CK-MB (ng/mL)	<2.0	<2.0	<2.0	<2.0	<2.0	<2.0	-
NT-proBNP (pg/mL)	6.25 ± 12.5	5.20 ± 10.3	25.00 ± 17.8	5.00 ± 11.2	2.00 ± 4.5	45.00 ± 32.5	0.528
CK (U/L)	114.75 ± 108.04	156.60 ± 99.36	244.60 ± 202.47	87.80 ± 27.83	294.40 ± 85.48	207.33 ± 50.0	0.591
D-Dimer (μg/mL)	<0.02	<0.02	<0.02	<0.02	<0.02	<0.02	-

* *Comparison between groups*.

**Figure 2:**
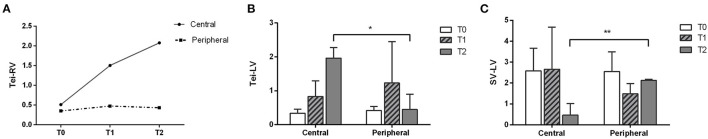
Changes in right ventricular **(A)** and left ventricular **(B)** Tei index and left ventricular stroke volume **(C)** in the two cannulation groups revealed a greater impact of central cannulation on ventricular function (*p < 0.05, **p < 0.01).

Echocardiographic data are shown in Table 2. Although the cannulation strategy did not affect the level of left ventricular Tei index (Tei-LV), a significant difference was observed in the level of right ventricular Tei index (Tei-RV) between the two cannulation groups (*p* = 0.016, Figure 2A). In the central cannulation group, both Tei-LV and Tei-RV increased over time, with Tei-LV at T2 being significantly higher than that at T0 (*p* < 0.001) and T1 (*p* = 0.004), suggesting impaired cardiac function under CPB. Tei-LV in the central cannulation group was even higher than in the peripheral cannulation group at T2 (1.96 ± 0.31 vs. 0.45 ± 0.19, respectively; *p* = 0.028, Figure 2B). Similarly, the levels of left ventricular stroke volume (SV-LV) between the two groups were not affected by the cannulation strategy (*p* = 0.854). However, SV-LV in the central cannulation group was significantly lower than in the peripheral cannulation group at T2 (0.46 ± 0.55 vs. 2.13 ± 0.05, respectively; *p* = 0.008, Figure 2C), indicating that central cannulation had a pronounced adverse impact on left cardiac function in addition to the changes in Tei-LV. There were insignificant difference between the two groups in other parameters, such as umbilical artery pulsation index, umbilical artery resistance index, heart rate, and blood pressure.

**Table 2:** Hemodynamic parameters.

	**Central cannulation**			**Peripheral cannulation**			*P*-value^*^
	**T0**	**T1**	**T2**	**T0**	**T1**	**T2**	
Tei-LV	0.34 ± 0.12	0.83 ± 0.46	1.96 ± 0.31	0.42 ± 0.12	1.23 ± 1.22	0.45 ± 0.19	0.111
Tei-RV	0.51 ± 0.14	1.50 ± 1.52	2.08 ± 1.1	0.35 ± 0.11	0.48 ± 0.15	0.43 ± 0.12	0.016
SV-LV	2.59 ± 1.08	2.66 ± 2.01	0.46 ± 0.55	2.55 ± 0.94	1.48 ± 0.49	2.13 ± 0.05	0.854
SV-RV	5.44 ± 4.74	2.56 ± 1.44	2.40 ± 2.76	2.81 ± 0.56	1.97 ± 0.11	2.75 ± 0.58	0.997
Umbilical artery-PI	0.94 ± 0.33	1.24 ± 0.30	1.18 ± 0.41	1.04 ± 0.08	1.19 ± 0.55	0.96 ± 0.3	0.720
Umbilical artery-RI	0.59 ± 0.12	0.65 ± 0.10	0.69 ± 0.13	0.63 ± 0.03	0.66 ± 0.22	0.60 ± 0.13	0.854
HR (bpm)	148.0 ± 6.25	86.5 ± 19.09	58.0 ± 26.87	134.3 ± 17.33	98.3 ± 29.42	97.8 ± 26	0.270
BP (mmHg)	43.25 ± 15.82	29.00 ± 14.65	28.33 ± 10.69	44.25 ± 2.06	21.67 ± 4.93	40.33 ± 10.41	0.709

* *Comparison between groups*.

**Figure 3:**
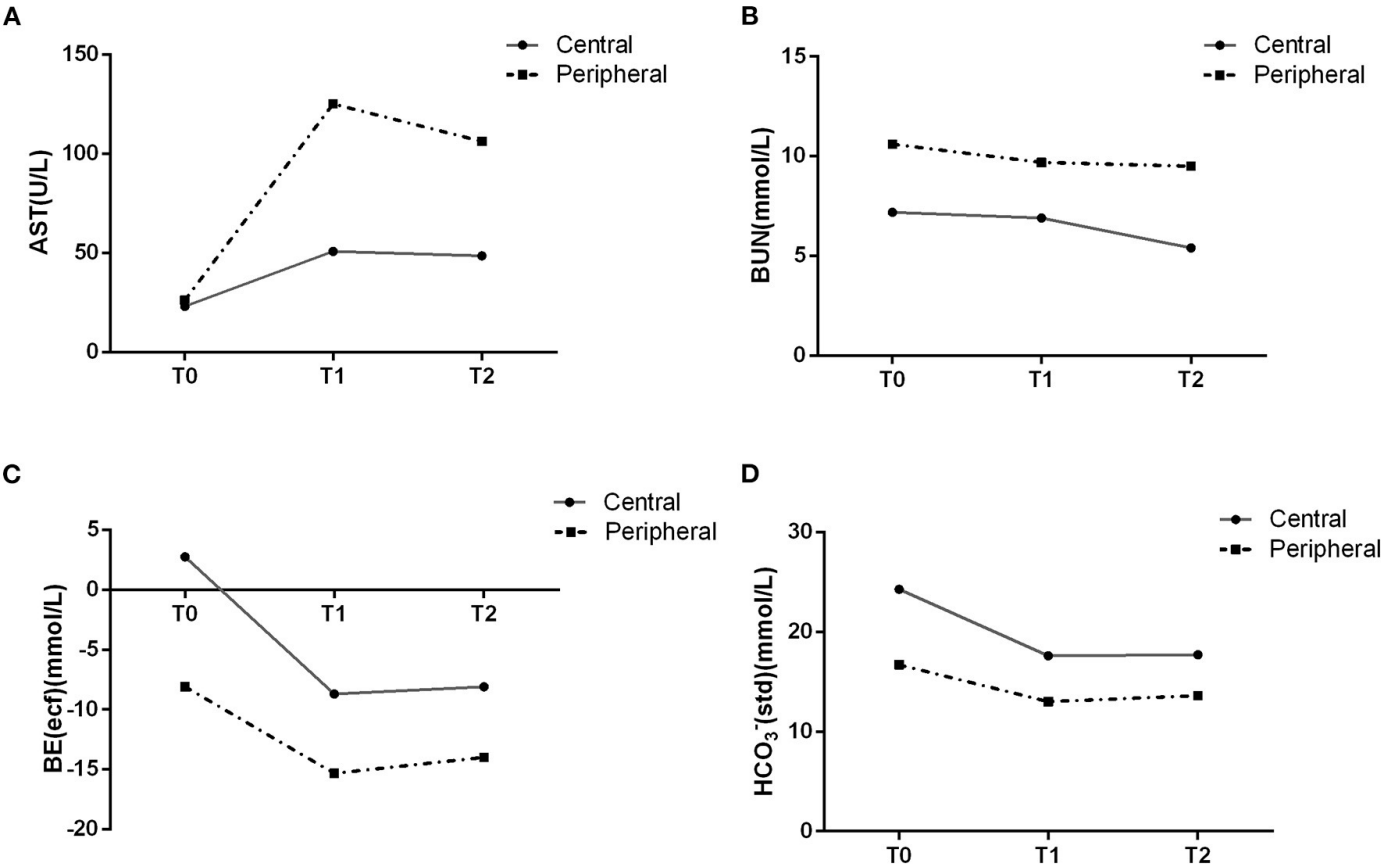
AST and BUN levels in the blood suggested an effect of peripheral cannulation on organ perfusion **(A,B)**. Changes in gas analysis are shown for the two cannulation groups **(C,D)**.

Liver function is shown in Table 3. Generally, there were no significant differences between the two groups except in AST levels (*p* = 0.047) and AST/ALT ratio (*p* = 0.024). Values of liver injury markers (AST, ALT and AST/ALT ratio) exhibited an increasing trend but remained stable at T1 and T2, suggesting limited impacts of fetal CPB on liver function. At T2, the elevation in AST (*p* = 0.017) and AST/ALT (*p* = 0.025) level was significantly lower in the central cannulation group (Figure 3A). These results indicate more severe liver injury in the peripheral cannulation group.

**Table 3:** Liver function tests.

	**Central cannulation**			**Peripheral cannulation**			*P*-value^*^
	**T0**	**T1**	**T2**	**T0**	**T1**	**T2**	
TP (g/L)	31.63 ± 8.42	27.42 ± 7.1	39.78 ± 16.76	27.80 ± 2.57	28.80 ± 8.11	26.50 ± 3.52	0.180
ALB (g/L)	21.28 ± 4.63	18.36 ± 9.31	20.34 ± 8.68	18.40 ± 1.14	17.65 ± 1.76	15.50 ± 1.92	0.259
Glo (g/L)	10.35 ± 3.96	9.06 ± 5.03	19.44 ± 15.26	9.40 ± 2.59	11.15 ± 6.88	11.00 ± 1.75	0.451
A/G	2.15 ± 0.42	4.57 ± 7.15	2.06 ± 2.74	2.12 ± 0.75	1.95 ± 0.85	1.42 ± 0.14	0.435
ALT (U/L)	15.00 ± 8.17	29.20 ± 26.58	30.80 ± 19.58	17.20 ± 4.44	37.40 ± 44.8	17.00 ± 5.2	0.908
AST (U/L)	23.00 ± 8.04	50.80 ± 27.96	48.60 ± 21.08	26.20 ± 12.79	125.20 ± 117.44	106.33 ± 29.37	0.047
AST/ALT	1.67 ± 0.52	2.57 ± 2.34	2.01 ± 1.61	1.71 ± 1.03	4.72 ± 4.13	6.73 ± 3.02	0.024
STB (μmol/L)	5.20 ± 2.95	9.26 ± 3.22	10.98 ± 9.96	7.48 ± 3.42	19.63 ± 24.56	7.83 ± 3.2	0.503
AMY (U/L)	40.75 ± 0.96	50.60 ± 20.42	52.40 ± 21.1	41.40 ± 0.55	41.20 ± 1.92	41.67 ± 1.53	0.212
CHOL (mmol/L)	1.53 ± 0.25	1.38 ± 0.35	1.36 ± 0.41	1.63 ± 0.22	1.13 ± 0.56	1.63 ± 0.31	0.779
Glu (mmol/L)	6.43 ± 2.88	10.93 ± 3.58	9.76 ± 4.64	9.07 ± 5.62	7.74 ± 4.16	4.56 ± 2.63	0.267

* *Comparison between groups*.

As shown in Table 4, the trend was toward decreasing levels of BUN across T0, T1, and T2 in both groups, with levels in the peripheral cannulation group significantly higher than in the central cannulation group (*p* = 0.018, Figure 3B). Moreover, the BUN/Cr ratio exhibited an increasing trend in the peripheral cannulation group, but decreased in the central cannulation group, suggesting severely interrupted renal perfusion in the peripheral group.

**Table 4:** Renal function test.

	**Central cannulation**			**Peripheral cannulation**			*P-*value^*^
	**T0**	**T1**	**T2**	**T0**	**T1**	**T2**	
TCa (mmol/L)	3.28 ± 0.26	2.35 ± 0.78	3.1 ± 0.43	2.55 ± 0.69	2.62 ± 0.48	3.09 ± 0.26	0.488
P (mmol/L)	2.34 ± 0.56	3.04 ± 0.85	3.04 ± 0.95	2.38 ± 0.59	3.22 ± 1.52	3.47 ± 0.78	0.564
BUN (mmol/L)	7.18 ± 3.02	6.97 ± 5.44	5.41 ± 0.86	10.76 ± 2.33	9.68 ± 3.03	9.51 ± 3.24	0.018
Cr (μmol/L)	148.50 ± 88.48	106.20 ± 61.1	91.00 ± 63.75	110.60 ± 41.6	108.20 ± 59.75	112.67 ± 41.19	0.846
BUN/Cr	18.20 ± 9.52	16.20 ± 15.87	12.67 ± 3.51	24.00 ± 16.79	27.20 ± 15.17	34.00 ± 18.08	0.037

* *Comparison between groups*.

Results of fetal blood gas analyses are shown in Table 5. As the duration of CPB increased, the level of carbon dioxide (pCO_2_) generally decreased in both groups. At T2, the peripheral cannulation group had a substantially elevated level of lactic acid compared with that at T0 (*p* = 0.017) and T1 (*p* = 0.015). In addition, we noted a significantly lower level of BE-ecf in the peripheral cannulation group across all time points (*p* < 0.001, Figure 3C). Although both groups exhibited decreasing bicarbonate levels over time, the peripheral group had significantly lower HCO3^−^act and HCO3^−^std across all time points (Figure 3D), indicating an influence of cannulation strategy on actual bicarbonate (*p* = 0.003) and standard bicarbonate (*p* = 0.001). Collectively, these results suggest a higher degree of metabolic acidosis and a more disrupted internal environment in the peripheral cannulation group.

**Table 5:** Blood gas analysis.

	**Central cannulation**			**Peripheral cannulation**			*P*-value^*^
	**T0**	**T1**	**T2**	**T0**	**T1**	**T2**	
pH	7.22 ± 0.06	7.42 ± 0.11	7.38 ± 0.17	7.14 ± 0.15	7.23 ± 0.17	7.31 ± 0.1	0.054
pO_2_ (mmHg)	20.00 ± 2.94	37.60 ± 28.84	37.40 ± 19.83	26.80 ± 8.35	41.60 ± 15.61	25.67 ± 0.58	0.964
pCO_2_ (mmHg)	77.40 ± 14.02	28.20 ± 12.03	31.58 ± 13.52	63.94 ± 20.78	31.46 ± 18.34	26.27 ± 8.8	0.408
Lac (mmol/L)	2.40 ± 0.71	6.46 ± 1.78	6.58 ± 4.14	3.21 ± 2.2	5.90 ± 4.33	10.00 ± 1.83	0.303
BE (ecf) (mmol/L)	2.75 ± 3.75	−8.7 ± 5.17	−8.1 ± 4.93	−8.18 ± 3.87	−15.32 ± 6.7	−14 ± 0.2	<0.001
BE (B) (mmol/L)	12.78 ± 25.03	1.02 ± 18.64	1.12 ± 17.73	19.24 ± 23.83	12.56 ± 22.94	31.90 ± 0.56	0.057
Hct (%)	33.00 ± 8.29	23.00 ± 10.86	22.40 ± 9.45	31.20 ± 6.5	17.00 ± 3.54	20.67 ± 3.06	0.310
${\text{HCO}}_{3}^{-}$act (mmol/L)	30.53 ± 3.6	15.82 ± 6.54	17.06 ± 4.38	20.76 ± 2.14	12.28 ± 5.69	12.33 ± 1.48	0.003
${\text{HCO}}_{3}^{-}$std (mmol/L)	24.43 ± 3.23	17.66 ± 3	17.76 ± 3.84	16.74 ± 3.2	13.02 ± 4.75	13.60 ± 0.3	0.001
SO_2_ (%)	24.75 ± 4.92	55.00 ± 32.37	59.20 ± 36.95	35.20 ± 22.08	62.40 ± 24.86	43.33 ± 8.5	0.949

* *Comparison between groups*.

## Discussion

In this study, we comprehensively assessed changes in cardiac function, hemodynamics and organ perfusion for central cannulation vs. peripheral cannulation in a fetal sheep CPB model. Overall, fetal sheep from both groups had stable hemodynamics during the entire 60 min CPB procedure. However, peripheral cannulation was associated with more side effects related to inadequate flow and organ perfusion, while central cannulation had greater adverse impacts on ventricular function. Therefore, when establishing a fetal CPB model, central cannulation might be preferred over peripheral cannulation in terms of maintaining relatively stable perfusion for peripheral organs, including the placenta.

We made several improvements in surgical technique to establish our fetal sheep CPB model. Fetal sheep are physiologically comparable to a human fetuses (19, 20) but with some important differences in cardiac anatomy. A sheep's heart lies at the median position, with the main pulmonary artery and right ventricle being fully exposed after median sternotomy. However, as the right atrium and superior vena cava are hidden in the lower position, it is difficult to expose these structures to establish CPB using the central cannulation technique. Because of the existence of the ductus arteriosus in the fetal circulatory system, cannulation of the main pulmonary artery is often used for fetal heart cannulation (9, 21, 22). In addition, the right atrium is fragile and vulnerable during surgical procedures. Therefore, we have made several modifications to surgical technique to improve the model. First, we performed longitudinal incision only on the upper-middle portion of the pericardium for better exposure while maintaining the integrity of the lower half to prevent an upturned left ventricle. This incision also rendered the heart less movable. Secondly, the hanging position for right pericardium was proximal to the reflex of the superior pericardial vena cava, which is better for exposure of the right atrium. As for pulmonary artery cannulation, we placed a purse-string suture with 7-0 prolene, followed by puncture at the center of the purse with a syringe needle (20 mL syringe). Then we inserted a 6 Fr cannula into the main pulmonary artery cannula while pulling out the needle. This technique prevents bleeding that result from direct incision of the main pulmonary artery. Since the superior vena cava is located at a deeper position, it is difficult to adjust the orientation of cannulas when the flow is unstable in a right-angle cannulation (23). We therefore prefer a right atrium cannulation with straight-tip cannulas to guarantee adequate venous drainage. When placing a purse-string suture on the right atrium, we used 7-0 prolene and a pericardium buttress for each stitch to minimize injury to right atrial tissue. All these techniques helped to improve the fetal CPB model.

Peripheral cannulation technology is mainly applied in ECMO and in recent years has been increasingly adopted in thoracoscopic or redo cardiac surgery (24, 25). The most common vessels for peripheral cannulation include the femoral artery and vein, followed by the axillary artery, carotid artery and jugular vein (26, 27). Different cannulation strategies can lead to variations in hemodynamics, which can alter organ and tissue perfusion depending on the choice of cannulation site (28, 29). Central cannulation is more analogous to physiological circulation and can provide sufficient flows for extracorporeal circulation. Moreover, cannulation at the ascending aorta with its larger diameter is unlikely to cause aortic stenosis. However, central cannulation requires median sternotomy, which is more invasive and can cause excessive bleeding. Some reports point out that patients undergoing central cannulation have significantly higher rates of infection, blood product usage and reoperation compared with those undergoing peripheral cannulation (30, 31). In addition, peripheral cannulation is less invasive and can be used in many different clinical scenarios, facilitating, for example, convenient transfer of extracorporeal equipment. However, sufficient venous drainage cannot be guaranteed in peripheral cannulation, and flow has to be constantly adjusted, which may lead to complications related to inadequate perfusion to peripheral organs and limbs (32).

The choice of cannulation strategy can greatly affect ventricular function. In our study, we observed comparable myocardial enzyme levels but different ventricular functional parameters in the two cannulation groups. Compared to the peripheral group, the central cannulation group had a significantly higher right ventricular Tei index. The echocardiographic results from the central cannulation group also showed a significantly higher left ventricular Tei index and lower left ventricular stroke volume at 60 min after CPB, suggesting an adverse impact of central cannulation on cardiac function. There could be several reasons for this finding: the central cannulation group (1) required a longer operation time, with longer exposure of the heart to room air; (2) had insufficient drainage and a relatively low volume during CPB, which could lead to cardiac insufficiency because of the Frank-Starling mechanism; and (3) required cannulation that could partially compress the heart, resulting in cardiac insufficiency, a problem not usually encountered in peripheral cannulation as the cannula is placed far away from the heart.

Although most indicators of liver and renal function were comparable between the two cannulation groups, a few (AST, BUN level and AST/ALT, BUN/Cr ratio) were significantly higher for peripheral than for central cannulation. This finding likely suggests the presence of inadequate organ perfusion and is consistent with changes observed in humans undergoing peripheral cannulation (30). For peripheral cannulation, the susceptibility to unstable flow after establishing extracorporeal circulation and the need to frequently adjust pipeline position (to maintain the rate of blood flow) could result in insufficient organ perfusion.

There were several limitations in our study, including the small sample size and limited number of time points. Future experiments with larger sample sizes and more time points may yield further insight into the underlying changes that occur during fetal CPB.

## Conclusion

Both central and peripheral cannulation for CPB are feasible in the fetal sheep. Central cannulation causes more cardiac injury and has a greater impact on ventricular function. Peripheral cannulation has fewer adverse effects on cardiac function but more on organ perfusion and dysfunction.

## Data Availability Statement

The raw data supporting the conclusions of this article will be made available by the authors, without undue reservation.

## Ethics Statement

All experiments are approved by the Animal Welfare and Ethics Committee of Guangdong Provincial People's Hospital (No. GDREC2019516A), and the experiments complied with The Guide for the Care and Use of Laboratory Animals (Association for Assessment and Accreditation of Laboratory Animal Care International, AAALACi).

## Author Contributions

CZ and YT: concept and design. MT and BH: data analysis and interpretation. XL: drafting article. JC: approval of article. YT: critical revision of article. JZ and CZ: funding. WP and WW: data collection. QJ: statistics. All authors contributed to the article and approved the submitted version.

## Funding

This work was supported by National Key Research and Development Program (2018YFC1002600), Natural Science Foundation of Guangdong (2018A030313785), Science and Technology Planning Project of Guangdong Province (2019B020230003, 2017B090904034, 2017B030314109, and 2018B090944002), and Guangdong Peak Project (DFJH201802, DFJH2019, and KJ012019446).

## Conflict of Interest

The authors declare that the research was conducted in the absence of any commercial or financial relationships that could be construed as a potential conflict of interest.

## Publisher's Note

All claims expressed in this article are solely those of the authors and do not necessarily represent those of their affiliated organizations, or those of the publisher, the editors and the reviewers. Any product that may be evaluated in this article, or claim that may be made by its manufacturer, is not guaranteed or endorsed by the publisher.

## Abbreviations

LV-IVCT: Left ventricular isovolumic contraction time
LV-IVRT: left ventricular isovolumic relaxation time
RV-IVCT: right ventricular isovolumic contraction time
RV-IVRT: right ventricular isovolumic relaxation time
AVD: aortic valve diameter
LVOT-VTI: left ventricular outflow tract velocity time integral
PVD: pulmonary valve diameter
RVOT-VTI: right ventricular outflow tract velocity time integral
Umbilical artery-PI: umbilical artery pulsation index
cTnI: troponin
Myo: myoglobin
NT-proBNP: N-terminal pro B type natriuretic peptide
CK: creatine kinase
ALT: alanine aminotransferase
AST: aspartate aminotransferase
TP: total serum protein
ALB: serum albumin
Glo: globulin
AMY: amylase
CHOL: total cholesterol
STB: serum total bilirubin, tCa, total calcium
BUN: blood urea nitrogen
Cr: creatinine
P: serum phosphorus
pO_2_: partial pressure of oxygen
pCO_2_: partial pressure of carbon dioxide
Lac: lactic acid
BE-ecf: base excess in the extracellular fluid compartment
BE-B: base excess
Hct: hematocrit
HCO3^−^act: actual bicarbonate
HCO3^−^std: standard bicarbonate
SO_2_: oxygen saturation.
